# The *Crocus sativus* Compounds *trans*-Crocin 4 and *trans*-Crocetin Modulate the Amyloidogenic Pathway and Tau Misprocessing in Alzheimer Disease Neuronal Cell Culture Models

**DOI:** 10.3389/fnins.2019.00249

**Published:** 2019-03-26

**Authors:** Ioanna Chalatsa, Demetrios A. Arvanitis, Nikolaos Stavros Koulakiotis, Athina Giagini, Alexios Leandros Skaltsounis, Zeta Papadopoulou-Daifoti, Anthony Tsarbopoulos, Despina Sanoudou

**Affiliations:** ^1^Clinical Genomics and Pharmacogenomics Unit, 4th Department of Internal Medicine, Medical School, National and Kapodistrian University of Athens, Athens, Greece; ^2^Molecular Biology Division, Center for Basic Research, Biomedical Research Foundation, Academy of Athens, Athens, Greece; ^3^GAIA Research Center, Bioanalytical Department, The Goulandris Natural History Museum, Athens, Greece; ^4^Department of Pharmacognosy and Natural Product Chemistry, Faculty of Pharmacy, National and Kapodistrian University of Athens, Athens, Greece; ^5^Department of Pharmacology, Medical School, National and Kapodistrian University of Athens, Athens, Greece; ^6^Center for New Biotechnologies and Precision Medicine, Medical School, National and Kapodistrian University of Athens, Athens, Greece

**Keywords:** natural compounds, beta-amyloid pathway, tau phosphorylation, Alzheimer’s disease, *trans*-crocin 4, *trans*-crocetin, *Crocus sativus*, neurodegenerative diseases

## Abstract

*Crocus sativus* L. natural compounds have been extensively used in traditional medicine for thousands of years. Recent research evidence is now emerging in support of its therapeutic potential for different pathologies including neurodegenerative diseases. Herein, the *C. sativus* L. natural compounds *trans*-crocin 4 and *trans*-crocetin were selected for in depth molecular characterization of their potentially protective effects against Alzheimer’s Disease (AD), utilizing two AD neuronal cell culture models (SH-SY5Y overexpressing APP and PC12 expressing hyperphosphorylated tau). Biologically relevant concentrations, ranging from 0.1 μM to 1 mM, applied for 24 h or 72 h, were well tolerated by differentiated wild type SH-SY5Y and PC12 cells. When tested on neuronally differentiated SH-SY5Y-APP both *trans*-crocin 4 and *trans*-crocetin had significant effects against amyloidogenic pathways. *Trans*-crocin 4 significantly decreased of β-secretase, a key enzyme of the amyloidogenic pathway, and APP-C99, while it decreased γ-secretases that generate toxic beta-amyloid peptides. Similarly, *trans*-crocetin treatment led to a reduction in β- and γ-secretases, as well as to accumulation of cellular AβPP. When tested on the neuronally differentiated PC12-htau cells, both compounds proved effective in suppressing the active forms of GSK3β and ERK1/2 kinases, as well as significantly reducing total tau and tau phosphorylation. Collectively, our data demonstrate a potent effect of *trans*-crocin 4 and *trans*-crocetin in suppressing key molecular pathways of AD pathogenesis, rendering them a promising tool in the prevention and potentially the treatment of AD.

## Introduction

*Crocus sativus* L. (saffron), has been extensively used for over 3,000 years in Iran, India, China, Spain, Italy, and Greece for culinary, coloring, cosmetic and medicinal purposes. In traditional medicine it has been considered for tens of different illnesses, while more recently, studies are suggesting potential antitumor, anticoagulant, antiatherogenic, antihypertensive and several other beneficial properties (Bathaie and Mousavi, 2010; Mancini et al., 2014). Of particular interest is the increasing evidence of saffron’s effects on the central nervous system (CNS). For example, saffron has been shown to have anticonvulsant and antidepressant effects, with the latter being comparable to imipramine and fluoxetine in treating patients with mild to moderate depression (Akhondzadeh et al., 2004, 2005; Hosseinzadeh and Sadeghnia, 2005; Noorbala et al., 2005; Hosseinzadeh and Sadeghnia, 2007). It also promotes cognitive functions in adult rodents previously exposed to amnestic agents (Zhang et al., 1994; Sugiura et al., 1995a; Zheng et al., 2007). Saffron also inhibited TNFR-induced apoptosis of neuronally differentiated PC12 cells (Soeda et al., 2001), and protected neurons from the neurotoxic activity of 6-hydroxydopamine hydrobromide (Ahmad et al., 2005).

However, saffron has multiple constituents, including polar carotenoids (crocins) that are mono-, di-, and triglucosyl esters of crocetin, a polyene dicarboxylic acid (8,8′- diapocarotene-8,8′-dioic acid), small amounts of monoterpene aldehydes, like picrocrocin and safranal, and flavonoids (quercetin and kaempferol) (Tarantilis et al., 1995; Koulakiotis et al., 2012). Crocins and crocetin, which are present in high concentration and primarily responsible for the color of saffron, appear to “drive” many of its neurological effects. For example, crocin was shown to inhibit neuronal death (Soeda et al., 2001; Ochiai et al., 2004), protect against cerebral ischemia/reperfusion injury, and enhance long term potentiation, learning, recognition and memory in rats (Sugiura et al., 1994, 1995b; Abe and Saito, 2000; Pitsikas et al., 2007). The consensus of the majority of studies performed to date support the therapeutic potential of crocin in aging and age-related neurodegenerative disorders in which cognitive impairment is involved (Finley and Gao, 2017). Crocetin has been shown to increase dopamine and its metabolites, to inhibit alpha-synuclein aggregation and promote alpha-synuclein fibril dissociation, with significant potential in Parkinson and other αS aggregation related diseases (Ahmad et al., 2005; Inoue et al., 2017). Finally, both crocin and crocetin also have neuroprotective properties by reducing the production of neurotoxic molecules from activated microglia (Nam et al., 2010).

These findings on crocin and crocetin, instigated research on their potential against Alzheimer’s disease (AD). AD is the most common form of dementia in the elderly, with a current estimate of 47 million patients worldwide which is expected to reach 131 million by 2050 (Alzheimer’s Association, 2013; Prince et al., 2016). AD patients experience a gradual decline in their cognitive functions, including thinking, remembering, reasoning as well as behavior. The key AD pathogenetic mechanisms involve impairment of the amyloidogenesis and neurofibrillary tangles (NFTs) pathways (Duan et al., 2012). Although many therapeutic agents have been considered, several are being tested, and five have already been approved, there is still no effective cure for AD. As the pursuit of chemical compounds has been resulting in limited success, while two of the approved drugs for AD are naturally derived, extensive research efforts are now being directed toward the investigation of natural compounds (Howes and Perry, 2011). Recently, the intranasal application of an antibody in mice led to the reduction of beta-amyloid (Aβ) accumulation induced cytotoxicity (Ferreira et al., 2018).

Saffron demonstrated significant benefits in cognition after treating mild-to-moderate Alzheimer’s disease patients a clinical pilot trial. Strikingly, cognitive improvements were comparable to those under treatment with donepezil (Akhondzadeh et al., 2010). Crocin and crocetin were shown to protect against beta-amyloid (Aβ) induced cell death *in vitro* and/or *in vivo*, leading to improvement of Aβ induced memory impairment in rats (Asadi et al., 2015). Crocin inhibited Aβ aggregation and fibril formation (Papandreou et al., 2006; Ghahghaei et al., 2012), while crocetin stabilizes Aβ oligomers, inhibits Aβ fibril formation and destabilizes preformed fibrils (Ahn et al., 2011). In respect to tau related pathogenetic mechanisms, crocin reduced acrolein-induced tau phosphorylation and oxidative stress *in vivo* (Rashedinia et al., 2015). Furthermore, it suppressed tau aggregation and the formation of NFTs, while it increased the orderly microtubule formation *in vitro* (Zarei Jaliani et al., 2013; Karakani et al., 2015).

In light of these observations we focused on discovering the molecular effects of crocin and crocetin on the key pathways of AD development, namely amyloidogenesis and tau phosphorylation. We specifically selected the predominant component of crocin, *trans*-crocin 4, and the unique C20 apocarotenoid, *trans*-crocetin, as they have been directly associated with decreased Aβ_1-40_ aggregation in neuronally differentiated PC12 cells and increased Aβ_42_ degradation in human AD monocytes, respectively (Papandreou et al., 2006; Tiribuzi et al., 2017). This study was conducted on two, established cellular models of AD, namely neuronally differentiated SH-SY5Y and PC12 cells, overexpressing the amyloid precursor protein or hyper-phosphorylated tau, respectively (Sotiropoulos et al., 2008; Chatzistavraki et al., 2013). We demonstrate that treatment with *trans*-crocin 4 or *trans*-crocetin can reverse a series of protein changes observed in APP misprocessing and tau hyperphosphorylation, supporting their preventive and possible therapeutic potential against AD.

## Materials and Methods

### Natural Compounds

*Trans*-crocin 4 and *trans*-crocetin, both active components of *C. sativus* L., were extracted, separated and isolated from dried stigmas of saffron flowers, provided by the Cooperative De Safran Krokos (Kozani, Greece), by semi-preparative HPLC, as previously described (Koulakiotis et al., 2012). Both compounds were diluted in DMSO.

### Mass Spectrometric Analysis

Mass spectral analysis of *trans*-crocin 4 and *trans*-crocetin was carried out by electrospray (ESI) mass spectrometry (MS) in the positive-ion mode employing a Waters Premier quadrupole reflectron time-of-flight (QqTOF) instrument. Analyte solutions containing 0.1–1 mg/mL of *trans*-crocin 4 in H_2_O and *trans*-crocetin dissolved in pure methanol were directly infused at a flow rate of 5 μL/min with a Harvard Apparatus Pump II syringe pump (Holliston, MA, United States). Accurate mass measurements of the aforementioned crocus-derived bioactive compounds were performed on-line on the QqTOF high-resolution MS using the leucine-enkephaline standard as a lock mass, as previously described (Koulakiotis et al., 2015). Structure characterization of *trans*-crocin 4 and *trans*-crocetin was carried out by ESI MS in combination with low-energy CID/tandem MS of the sodiated [M+Na]^+^ precursor ions on the QqTOF mass spectrometer.

### Cell Culture and Differentiation

The human neuroblastoma SH-SY5Y and rat pheochromocytoma PC12 cell lines were used as well established neuronal models that can differentiate into neuron-like cells. Furthermore, SH-SY5Y-APP cells inducibly over-expressing APP695 (a kind gift of Dr. S. Efthimiopoulos, Faculty of Biology, University of Athens, Greece) (Chatzistavraki et al., 2013), and PC12-htau cells stably transfected with the human tau (htau; 3R/0N isoform) transgene and expressing hyperphosphorylated tau [a kind gift of Dr. I. Sotiropoulos, Life and Health Sciences Research Institute (ICVS), School of Health Sciences, University of Minho, Portugal] (Sotiropoulos et al., 2008) were used as *in vitro* models of AD. All cells were cultured in Dulbecco’s Modified Eagle’s Medium (DMEM) without L-glutamine, maintained at 37°C in a humidified 5% CO_2_ environment. For SH-SY5Y cells, the medium was supplemented with 10% (vol/vol) heat-inactivated Fetal Bovine Serum (FBS), 1% antibiotic/antimycotic (10,000 units/mL of penicillin, 10,000 μg/mL of streptomycin, 25 μg/mL of amphotericin B) and 1% L-alanyl-L-glutamine. For SH-SY5Y-APP cells, the SH-SY5Y culture medium contained in an additional 100 μg/mL of G418 (Gibco, Thermo Fisher Scientific Inc.). For PC12 cells, the DMEM without L-glutamine was supplemented with 5% (v/v) heat-inactivated Horse Serum (HS) and the cells were plated on collagen-treated flasks/plates (Fath et al., 2002). For PC12-htau cells the culture medium contained an additional 100 μg/ml of G418 (Gibco, Thermo Fisher Scientific Inc.). Differentiation of SH-SY5Y and SH-SY5Y-APP cells was achieved with the addition of all-*trans* retinal (Sigma-Aldrich Co.) to the culture media, to a final concentration of 10^−5^ M for 6 days. For the differentiation of PC12 and PC12-htau cells 0.75% FBS, 075% HS, 100 ng/ml 7S nerve growth factor (NGF; Invitrogen), 1% antibiotic/antimycotic and 1% L-alanyl-L-glutamine were added to the media for 7 days.

### Natural Products Cell Viability Assays

Differentiated SH-SY5Y or PC12 cells were exposed to *trans*-crocin-4 or *trans*-crocetin concentrations ranging from 0.1 μM to 1 mM, for 24 h or 72 h. The effect of the exposure on cell viability was evaluated with the 3-(4,5-di-methylthiazol-2-yl)-2,5-diphenyltetrazolium bromide assay (MTT; Sigma-Aldrich Co.), which is based on the cleavage of MTT by mitochondrial reductase and the absorbance of the resulting formazan crystals at 570 nm in an ELISA reader (ELx800; BioTek Instruments). Matched concentrations of DMSO were used as control and all experiments were performed at least three times.

### Immunofluorescence Studies

Cells were cultured on poly-D-lysine-coated glass coverslips, fixed in 3.7% paraformaldehyde for 20 min at 4°C, and incubated in blocking buffer (PBS containing 10% normal goat serum, 0.4% Triton X-100) for 1 h at room temperature (RT). The primary antibodies rabbit anti-MAP2 (1:400; Santa Cruz Biotechnology), mouse anti-NeuN (1:500; EMD Millipore) and mouse anti-TUJ1 (1:2000; Covance) were applied for overnight incubation. The secondary antibodies mouse Cy3 and rabbit Cy2 (Jackson ImmunoResearch, West Grove, PA, United States) were applied at 1:500 dilution, for 1 h at RT. The samples were mounted with Vectashield containing 4′,6-diamidino-2-phenylindole (DAPI) (Vector Laboratories, Burlingame, CA, United States) and analyzed with a Leica fluorescence microscope. The slides were photographed with an ORCA-Flash 4.0 digital color camera and the images processed with the ImageJ software (version 1.47v^1^).

### SDS PAGE and Immunoblotting

Cells were lysed in lysis buffer (50 mM Tris, pH 7.5, 150 mM NaCl, 2 mM EDTA, 1% Triton) supplemented with a mixture of protease inhibitors (P8340; Sigma-Aldrich Co.), incubated on ice for 30 min and centrifuged at 13,000 rpm for 5 min. The protein concentration was determined with the Bradford method (Bradford, 1976), using bovine serum albumin for the standard curve. All samples were analyzed by SDS-PAGE (Supplementary Table S1). GAPDH was used as loading control for the SH-SY5Y (Castaño and Kypta, 2008) and actin for the PC12 experiments (Chung et al., 2010). For the analysis of sAPPα the culture medium was collected and condensed with centrifugal filter units (Amicon; Millipore) at 4,000 rpm, 16.5% Tris-Tricine gels were employed, and the volume of the initial culture media was used in sample normalization and reciprocal sample volume loading. Incubation with primary antibodies (Supplementary Table S1) took place following protein transfer to nitrocellulose membranes (MACHEREY-NAGEL GmbH & Co). The nitrocellulose membranes were subsequently washed in 50 mM Tris-HCl, pH 7.5, 150 mM NaCl, and 0.05% Tween 20 and incubated with a peroxidase-conjugated anti-mouse (1:16,000 dilution; Sigma-Aldrich Co.) or anti-rabbit (1:10,000 dilution; BIO-RAD) secondary antibody. Protein signals were detected using electrogenerated chemiluminescence (ECL) reagents according to manufacturer’s recommendations (Thermo Fisher Scientific Inc.). The bands of interest from at least three different experiments were quantified using Image J software (version 1.47v^1^). The measurements for all phosphorylated forms were normalized to the total levels of the corresponding proteins.

### Statistical Analysis

All measurements were analyzed with descriptive statistics and the results presented as mean ± standard deviation (SD). The *p*-value threshold was set at ≤0.05. For the analysis of the viability assay results, the two curves were compared at a time with two-way analysis of variance (ANOVA) of the logarithm of viability, in each experiment, using the Akaike information criterion (AIC) approach. For the western blot analysis, the integrated densities of the western blot bands were quantified and analyzed by one-way ANOVA, followed by multiple comparisons *post hoc* test based on Holm–Sidak method, where it was appropriate.

## Results

### Mass Spectrometric Analysis and Structure Characterization of *trans*-Crocin 4 and *trans*-Crocetin

High-resolution ESI MS analysis of *trans*-crocin 4 and *trans*-crocetin in the positive-ion yielded an abundant [M+Na]^+^ pseudo-molecular ion. Further analysis of these [M+Na]^+^ ions by ESI MS/MS in the QqTOF high-resolution MS instrument afforded structural information on these isolated components as shown in Supplementary Figures S1, S2, respectively. In a previous study, the differentiation of positional crocetin glycoside isomers in a series of crocetin glycoconjugates was carried out by MALDI-RTOF MS and high-energy tandem MS (Koulakiotis et al., 2012). The structure confirmation of the aforementioned crocus-derived compounds afforded by the HRMS mass measurement accuracy, as well as the tandem MS pattern, is a prerequisite for the ensuing evaluation of their potentially protective effects against AD.

### Differentiation of Neuroblastoma and Pheochromocytoma Cell Lines to Neuron-Like Cells

**FIGURE 1:**
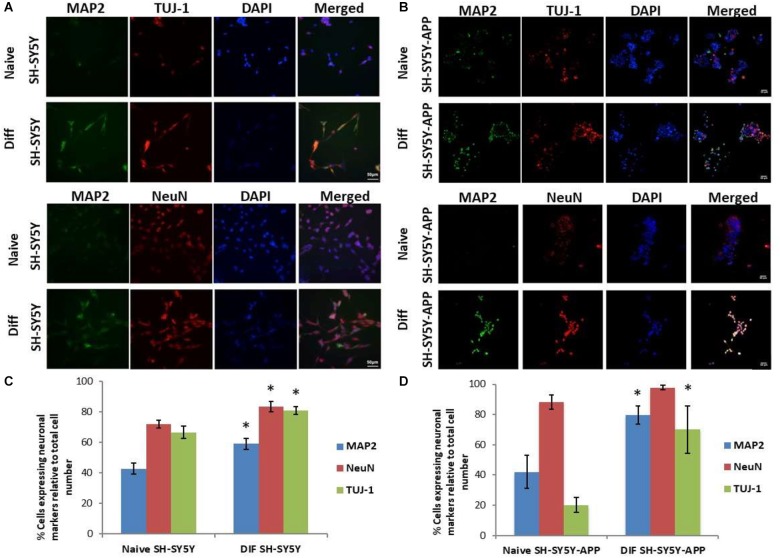
Expression of the neuronal markers MAP2, NeuN, and TUJ-1 in differentiated SH-SY5Y and SH-SY5Y-APP cells. **(A)** Representative images of naive and differentiated (at 6 days) SH-SY5Y, and **(B)** SH-SY5Y-APP cells. Diagrammatic presentation of quantified protein expression in SH-SY5Y **(C)**, and SH-SY5Y-APP cells **(D)** (^∗^*p* < 0.05, *t*-test, *n* = 3).

**FIGURE 2:**
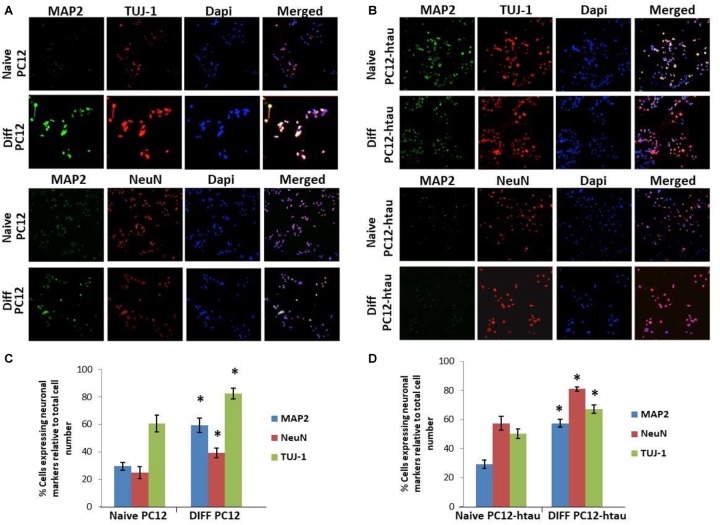
Expression of the neuronal markers MAP2, NeuN, and TUJ-1 in differentiated PC12 and PC12-htau cells. **(A)** Representative images of naive and differentiated (at 7 days) PC12, and **(B)** PC12-htau cells. Diagrammatic presentation of quantified protein expression in PC12 **(C)**, and PC12-htau cells **(D)** (^∗^*p* < 0.05, *t*-test, *n* = 3).

The initial step toward evaluating the molecular effects of *trans*-crocin 4 and *trans*-crocetin was the establishment of a suitable *in vitro* screening model. Toward this end the neuroblastoma, wild type SH-SY5Y and SH-SY5Y-APP cell lines were incubated with all-*trans* retinal for 6 days, while the pheochromocytoma wild type PC12 and PC12-htau cell lines were incubated with NGF for 7 days, to enable differentiation to neuron-like cells. The efficiency of the differentiation process was evaluated by microscopical examination of cell morphology and the expression of neuronal specific protein markers. The successful differentiation was determined by a decrease in proliferation rates, the outgrowth of neurites (Supplementary Figure S3) (Cheung et al., 2009; Dwane et al., 2013), and the assessment of three neuronal markers, namely: (a) microtubule-associated protein 2 (MAP2), that stabilizes microtubules and is critical for neurite outgrowth and dendrite development (Goedert et al., 1991; Presgraves et al., 2004; Bond et al., 2012), (b) nuclear marker NeuN, a marker for post-mitotic neurons and maturation (Cheung et al., 2009; Agholme et al., 2010), and (c) neuron specific β-Tubulin III (TUJ-1), which is almost exclusively expressed in neurons and is a marker for differentiation and decreased proliferation (Figure 1, 2) (Constantinescu et al., 2007; Bell et al., 2013; Dwane et al., 2013). Their expression levels were statistically significantly increased, confirming the successful differentiation of all four cell types to neuron-like cells (Figure 1, 2). We therefore, proceeded to use the differentiated wild type SH-SY5Y and PC12 cells as an *in vitro* model of neuron-like cells, and the differentiated SH-SY5Y-APP and PC12-htau as cellular models of AD.

### *Trans*-Crocin 4 and *trans*-Crocetin Do Not Compromise Viability of Neuron-Like Cells

**FIGURE 3:**
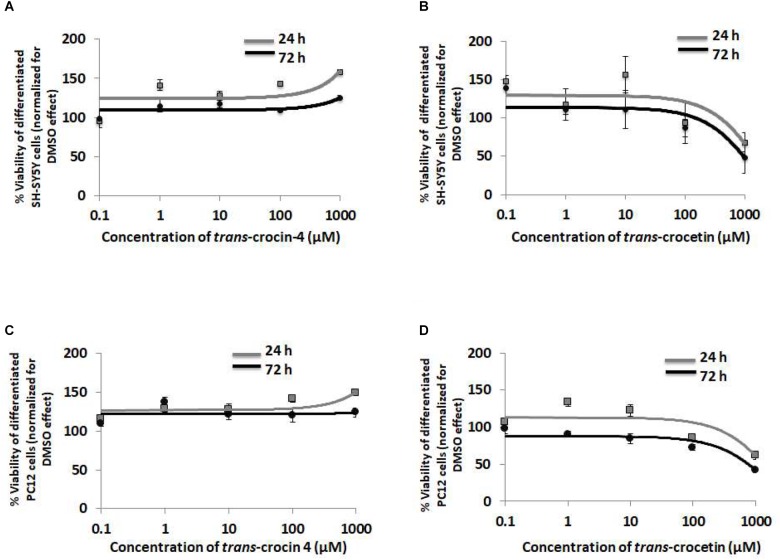
*Trans*-crocin 4 and *trans*-crocetin are well tolerated by neuron-like cells. Diagrammatic presentation of the MTT assay results for compound concentrations 0.1 μM to 1 mM, and incubation times 24 and 72 h, of differentiated SH-SY5Y **(A,B)** as well as PC12 **(C,D)** cells (*n* = 3). Cell viability is unaltered by *trans*-crocin 4 and *trans*-crocetin for concentrations up to 100 μM. The concentration axis is log scaled. The observations for the 24 h versus the 72 h incubation time period, for the same treatment and cell type were similar (*p* > 0.05, two-way ANOVA, *n* = 3).

To evaluate the biological tolerance of neuron-like cells for *trans*-crocin 4 and *trans*-crocetin, we exposed them to a biologically relevant of range of concentrations (0.1 μM to 1 mM) for different time periods (Grossi et al., 2013; Kostomoiri et al., 2013; Luccarini et al., 2014). Both compounds were considered well tolerated across the tested concentrations, at both 24 and 72 h post-treatment, with *trans*-crocin 4 even enhancing cell growth at the highest tested concentrations (Figure 3A,B). On the contrary the highest concentrations of *trans*-crocetin compromised to some extent cell viability (Figure 3B). These findings were fully reproduced in differentiated PC12 cells (Figure 3C,D), confirming that *trans*-crocin 4 and *trans*-crocetin do not compromise cell viability of neuron-like cells at the specific concentrations and incubation times.

### *Trans*-Crocin 4 and *trans*-Crocetin Modulate APP Processing

**FIGURE 4:**
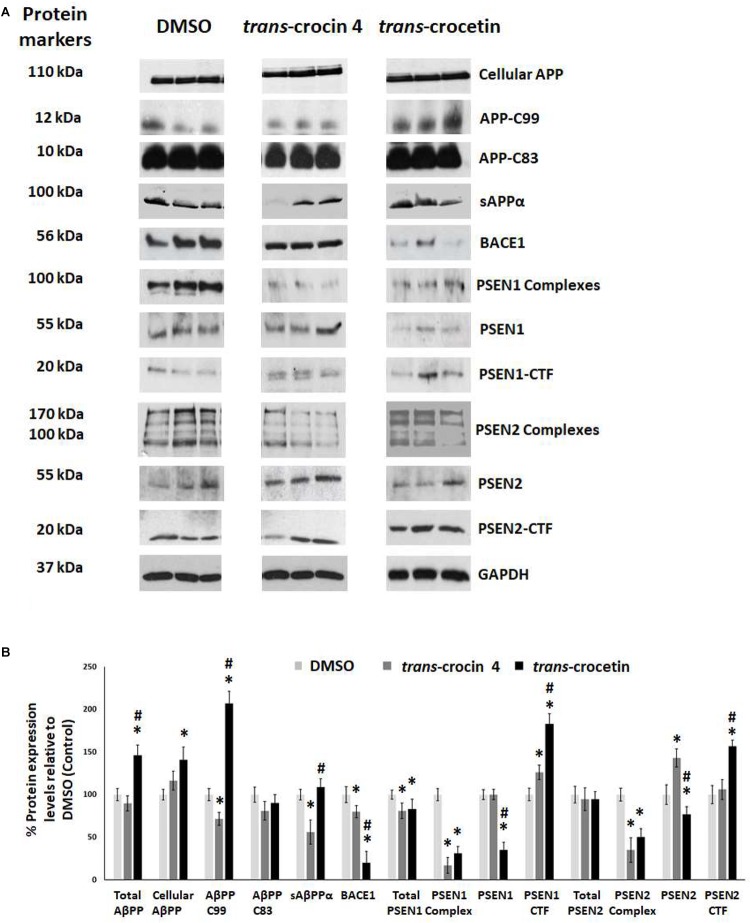
Western blot analysis of APP processing components after treatment of differentiated SH-SY5Y-APP cells with *trans*-crocin 4 or *trans*-crocetin. **(A)** Immunoblotting detection of cellular APP, APP-C99, APP-C83, sAPPα, BACE1, PSEN1 complexes, PSEN1, PSEN1-CTF, PSEN2 complexes, PSEN2, and PSEN2-CTF, with GAPDH as internal control of protein expression. **(B)** Diagrammatic presentation of the percent change in expression of protein levels following treatment relative to DMSO control. Total APP protein levels were based on the sum of the immunoblotting measurements of cellular APP, APP-C99, APP-C83, and sAPPα. (^∗^*p* < 0.05, for the comparison of test treatment versus DMSO in *post hoc* one-way ANOVA, *n* = 3; ^#^*p* < 0.05, for the comparison of *trans*-crocetin versus *trans*-crocin 4 in *post hoc* one-way ANOVA, *n* = 3).

Since APP processing is considered a central pathogenetic mechanism for amyloidogenesis and AD, we proceeded to assess the effect of *trans*-crocin 4 and *trans*-crocetin on the expression of its key molecular players. Differentiated SH-SY5Y-APP cells were treated with the maximal non-toxic concentration of each compound for 72 h (1 mM *trans*-crocin 4, 10 μM *trans*-crocetin) and compared against DMSO treatment. APP-C83 and sAPPα were used as markers of the non-amyloidogenic α-amyloid pathway processing, while APP-C99, β-secretase (BACE1) and the catalytic components of γ-secretase (PSEN1 or PSEN2) as markers of the amyloidogenic β-amyloid pathway (Haass and Selkoe, 2007; Wakabayashi and De Strooper, 2008; Haass et al., 2012; Zhang et al., 2013). PSEN1 and PSEN2 are the products of the respective full-length protein cleavage and amino-/carboxy- terminal fragment (NTF/CTF) heterodimerization (Alves da Costa et al., 2003; Laudon et al., 2004), and are also encountered in the form of multiprotein complexes (Hebert et al., 2003; Garcia-Ayllon et al., 2013). Each compound was compared to the DMSO treatment. *Trans*-crocin 4 significantly reduced total PSEN1 by 19%, and the PSEN1 and PSEN2 complexes by 81 and 65%, while it increased PSEN1-CTF (26%) and PSEN2 (43%) (Figure 4). The key molecules of the amyloidogenic pathway, BACE1 and APP-C99 were significantly reduced by 20 and 28%, respectively. The non-amyloidogenic pathway product sAPPα was also reduced, by 44%, while APP-C83 remained unaltered. *Trans*-crocetin dramatically reduced BACE1 (by 80%), total PSEN1 (by 17%), PSEN1 (by 65%) and PSEN2 (by 23%), as well as their complexes (by 69 and 50%, respectively), while it increased PSEN1- and PSEN2-CTF (by 83 and 57%, respectively). Meanwhile it significantly increased total APP by 46%, cellular APP by 41%, and APP-C99 by 107% (Figure 4).

Collectively, treatment with *trans*-crocin 4 decreased β-secretase (BACE1) and APP-C99, suggesting the suppression of the amyloidogenic pathway. Meanwhile it markedly decreased γ-secretases (PSEN1 and PSEN2 complexes) that generate toxic Aβ peptides. *Trans*-crocetin treatment led to a dramatic reduction of BACE1, as well as a significant reduction of PSEN complexes, all of which are key enzyme of the amyloidogenic pathway, yet it increased APP-C99. Consequently, *trans*-crocin 4 and *trans*-crocetin have significant, yet partly different effects on the molecular pathways implicated in amyloidogenesis, supporting a beneficial role against AD.

### *Trans*-Crocin 4 and *trans*-Crocetin Modulate Tau Phosphorylation

**FIGURE 5:**
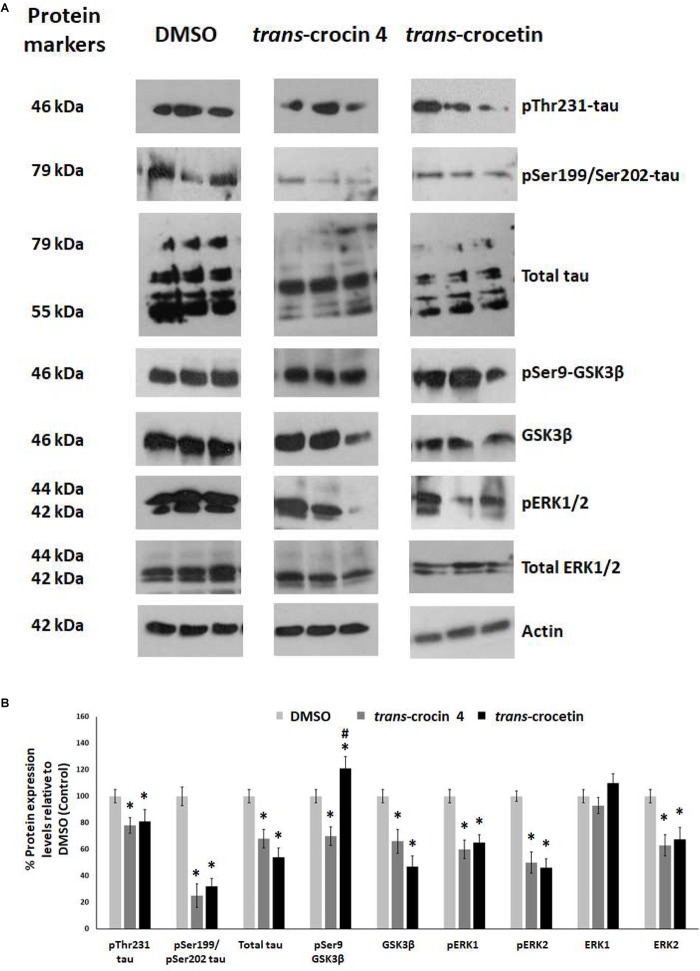
Western blot analysis of the tau phosphorylation pathway components after treatment of differentiated PC12-htau cells with *trans*-crocin 4 or *trans*-crocetin. **(A)** Immunoblotting detection of pThr231-tau, pSer199/Ser202-tau, total tau, pSer9-GSK3β, GSK3β, pERK1/2, total ERK1/2, with actin as internal control of protein expression. **(B)** Diagrammatic presentation of quantified protein expression (^∗^*p* < 0.05, for the comparison of test treatment versus DMSO in *post hoc* one-way ANOVA, *n* = 3; ^#^*p* < 0.05, for the comparison of *trans*-crocetin versus *trans*-crocin 4 in *post hoc* one-way ANOVA, *n* = 3).

Tau hyperphosphorylation is believed to be crucial in AD pathogenesis by promoting the formation of NFTs and ultimately neural loss. We have previously demonstrated that tau is hyperphosphorylated at Thr231 and Ser199/Ser202 in PC12-htau cells compared to PC12 cells (Chalatsa et al., 2018). We therefore proceeded to assess the effects of *trans*-crocin 4 and *trans*-crocetin on this molecular mechanism using differentiated PC12-htau cells expressing hyperphosphorylated human tau. The endpoints measured included tau phosphorylation (pThr231-tau and pSer199/Ser202-tau), as well as expression and activation of the tau kinases GSK3β and ERK1/2. The levels of the phosphorylated proteins were normalized against the corresponding total protein expression levels. Both GSK3β and ERK1/2 were found to be activated in control PC12-htau cells, in agreement with previous reports on AD (Figure 5) (Amadoro et al., 2006; Mazanetz and Fischer, 2007; Forlenza et al., 2011; Medina and Wandosell, 2011; Martin et al., 2013).

Treatment with *trans*-crocin 4 significantly reduced total tau levels (by 32%) and tau phosphorylation (pThr231 and pSer199/Ser202-tau by 22 and 75%, respectively). Significant downregulation was also observed for both active and inactive forms of GSK3β (total GSK3β by 34%, pSer9-GSK3β by 30%) and ERK1/2 (total ERK2 by 37%, pERK1 by 40%, pERK2 by 50%), with the exception of ERK1 which remained unaltered. A highly similar outcome was observed following treatment with *trans*-crocetin. In specific, it significantly reduced both total (by 46%) and phosphorylated tau (pThr231 by 19% and pSer199/Ser202 by 68%), as well as GSK3β (by 53%), ERK2 (by 37%), pERK1 (by 35%) and pERK2 (by 32%). The only distinct difference observed between *trans*-crocetin and *trans*-crocin 4 was the significant increase in the inactive pSer9-GSK3β by the former.

In addition, to eliminate the possibility the effects obtained for *trans*- crocin 4 and *trans*-crocetin on PC12-htau cells being related to the vector carrying the transgene, we assessed the effects of the two compounds on tau expression in wild type differentiated PC12 cells. Both compounds were shown to have highly similar effects in PC12 and PC12-htau cells (Supplementary Figure S4).

Overall, *trans*-crocin 4 and *trans*-crocetin proved effective in suppressing GSK3β and ERK1/2 kinases in PC12-htau cells, and significantly reducing the levels and phosphorylation of tau on the pThr231 and pSer199/Ser202 epitopes. In addition, *trans*-crocetin significantly increased the levels of inactive GSK3β.

## Discussion

Alzheimer’s disease is characterized by the aggregation of amyloid plaques and the formation of NFTs. Amyloid plaques result from impairments in the amyloid pathway. Physiologically, APP can be processes through the non-amyloidogenic pathway, where it is cleaved by the α-secretase to produce sAPPα and APP-C83 which is subsequently cleaved by the γ-secretase (catalytic subunits: PSEN1 or PSEN2), or the amyloidogenic pathway, where it is cleaved by β-secretase (BACE1) to give APP-C99 which is subsequently cleaved by γ-secretase into Aβ and AICD (Prox et al., 2012). The NFTs consist primarily of hyperphosphorylated and misfolded tau protein, a microtubule-associated protein with an otherwise important role in the assembly of the neuronal microtubules network. Activated (unphosphorylated) GSK3β and (phosphorylated) ERK1/2 tau kinases are implicated in the process and have been shown to contribute to their formation (Martin et al., 2013).

In search of new therapeutic approaches against AD and in light of recent observations pointing to a potentially beneficial effect of *trans*-crocin 4 and *trans*-crocetin, we focused on the investigation of their molecular effects on the amyloid and tau phosphorylation pathways. It is noteworthy, that changes in the APP and tau processing have also been described in Down’s syndrome (Lee et al., 2016), while tau alterations have been detected in a wide range of neurodegenerative disorders collectively called tauopathies (Williams, 2006), as well as in aging (Wharton et al., 2016). Consequently, a potential beneficial effect of *trans*-crocin 4 and *trans*-crocetin in these pathways, could be relevant and valuable in a number of different pathological settings.

Initially, we assessed the effects of *trans*-crocin 4 and *trans*-crocetin on wild type cell viability, using the well-established SH-SY5Y and PC12 cell lines. Both were well tolerated, with higher concentrations of *trans*-crocin 4 even enhancing cell proliferation. The highest tested biologically relevant and viability favorable doses were selected for in depth studies of the APP and the tau processing pathway using the established *in vitro* models of AD, SH-SY5Y-APP, and PC12-htau, respectively.

The treatment of neuronally differentiated SH-SY5Y-APP cells with *trans*-crocin 4 showed a significant decrease in BACE1, which represents a validated target for AD therapy due to its distinctive role in its pathogenesis (Jonsson et al., 2012). Its inhibition by various means has led to reduced Aβ burden and amyloid plaque deposition, in *in vitro* as well as *in vivo* models (Videira et al., 2014; Zhang et al., 2014). BACE1 is the beta secretase mediating the production of APP-C99 and ultimately Aβ, which is found to be significantly increased in the brains of AD patients (Rossner et al., 2006; Garcia-Ayllon et al., 2013). Notably, APP-C99 was also significantly downregulated following treatment with *trans*-crocin 4. Previously, selective neutralization of APP-C99 has been shown to reduce the production of Aβ peptides (Houacine et al., 2012). The production of Aβ from APP-C99 is mediated by γ secretase, whose catalytic components include PSEN1 and PSEN2. In our data, *trans*-crocin 4 led to multiple significant changes in the levels of PSEN1 and 2, as well as their CTF fragments and their complexes. The most impactful one would be anticipated to be the reduction of the latter (i.e., PSEN1 and PSEN2 complexes), as they have been found to be increased in the cerebrospinal fluid of AD patients (Garcia-Ayllon et al., 2013) and to generate the main source of the intracellular Aβ-pool (Sannerud et al., 2016), respectively. Targeting γ secretase has been considered a promising therapeutic approach, with various inhibitors and modulators being tested (Wolfe, 2012). In addition, we found that incubation of differentiated SH-SY5Y-APP cells with *trans*-crocin 4 significantly reduced sAPPα. Although sAPPα is a product of the non-amyloidogenic pathway, it has been found significantly increased in the cerebrospinal fluid of AD patients, with their levels closely associated with sAPPβ, and has therefore been proposed as a promising biomarker for the clinical diagnosis of AD (Lewczuk et al., 2008). Consequently, our findings taken as a whole, appear to suggest a potent effect of *trans*-crocin 4 toward the suppression of the amyloidogenic pathway.

The effects of *trans*-crocin 4 on the tau pathways were investigated using neuronally differentiated PC12-htau cells. We observed a statistically significant decrease in phosphorylated tau (based on the assessment of epitopes pThr231-tau and pSer199/Ser202-tau), as well as total tau. This decrease would be anticipated to reduce tau dissociation from microtubules and limit tau aggregation in AD (Martin et al., 2011). Treatment with *trans*-crocin 4 also significantly reduced the expression levels of the active pERK1, pERK2, and GSK3β, as well as the inactive ERK2 and pSer9-GSK3β forms (Kim and Choi, 2010; Spitzer et al., 2011; Martin et al., 2013). These effects could have a significant therapeutic potential, since increased levels of phosphorylated (active) ERK is associated with early tau deposition in neurons and glial cells in tauopathies (Ferrer et al., 2001), and GSK3β over-expression leads to apoptosis *in vitro*, neurodegeneration in transgenic mice and NFT formation in AD patients (Martin et al., 2011, 2013). Furthermore, active GSK3β has been shown induces tau hyperphosphorylation and neurofibrillary lesions (Cai et al., 2012). The sum of these findings would support a reduction in NFT formation.

Interestingly, the activation of GSK3β is also implicated in amyloidogenesis by inhibiting the secretory cleavage of APP, increasing the production of Aβ and leading to memory impairment in animal models (Forlenza et al., 2011). Its inactivation by *trans*-crocin 4, together with the downregulation of PSEN1 and PSEN2 complexes, provide strong evidence to support and explain the mechanisms of *trans*-crocin 4 inhibition of Aβ aggregation *in vitro* (Papandreou et al., 2006; Ghahghaei et al., 2012).

Treatment with *trans*-crocetin led to a statistically significant over-expression of total APP, and APP-C99 in SH-SY5Y-APP cells. In regards to γ-secretase, the PSEN1 and PSEN2 protein levels were significantly reduced, along with total PSEN1, PSEN1 and PSEN2, while the PSEN1-CTF and PSEN2-CTF were increased. Accumulation of APP-C99 could occur through increased APP levels (Winton et al., 2011), especially in combination with inhibition of γ-secretase (Lauritzen et al., 2012), which was indeed the case in our study. Paradoxically, although the protein levels of APP-C99 were significantly increased, BACE1 expression was down regulated. This could potentially be explained by a feedback inhibition mechanism affecting BACE1 expression. For example, it has been previously reported that high concentrations of Aβ can activate α4β2AChRs in rat hippocampal GABAergic interneurons (Jurgensen and Ferreira, 2010) and activation of α4β2AChRs can suppress BACE1 transcription through the ERK1-NFκB pathway (Wang et al., 2012). The reduced β-secretase expression together with the downregulated γ-secretase components could lead to inhibition of Aβ formation, which would explain previous observations showing that *trans*-crocetin inhibits Aβ fibril formation and destabilizes pre-formed Aβ fibrils *in vitro* (Ahn et al., 2011).

*Trans*-crocetin appeared to be effective in modulating the tau pathway, similarly to *trans*-crocin 4. Their effects were largely the same. In specific, both compounds significantly reduced total tau and tau phosphorylation, and promoted ERK1, ERK2, and GSK3β inactivation. Importantly, immunization studies in animal models indicate that reduction of intracellular levels of tau and phosphorylated tau can improve cognitive performance (Giacobini and Gold, 2013). Meanwhile GSK3β inhibitors are in clinical trials as a promising treatment against AD, and GSK3 inhibition has been shown to ameliorate Aβ pathology in animal models of AD (Sofola et al., 2010; del Ser et al., 2013; Georgievska et al., 2013; Prati et al., 2015). Consequently, *trans*-crocetin appears to have a beneficial effect on multiple AD therapeutic targets at the same time, therefore representing a promising compound against AD.

**FIGURE 6:**
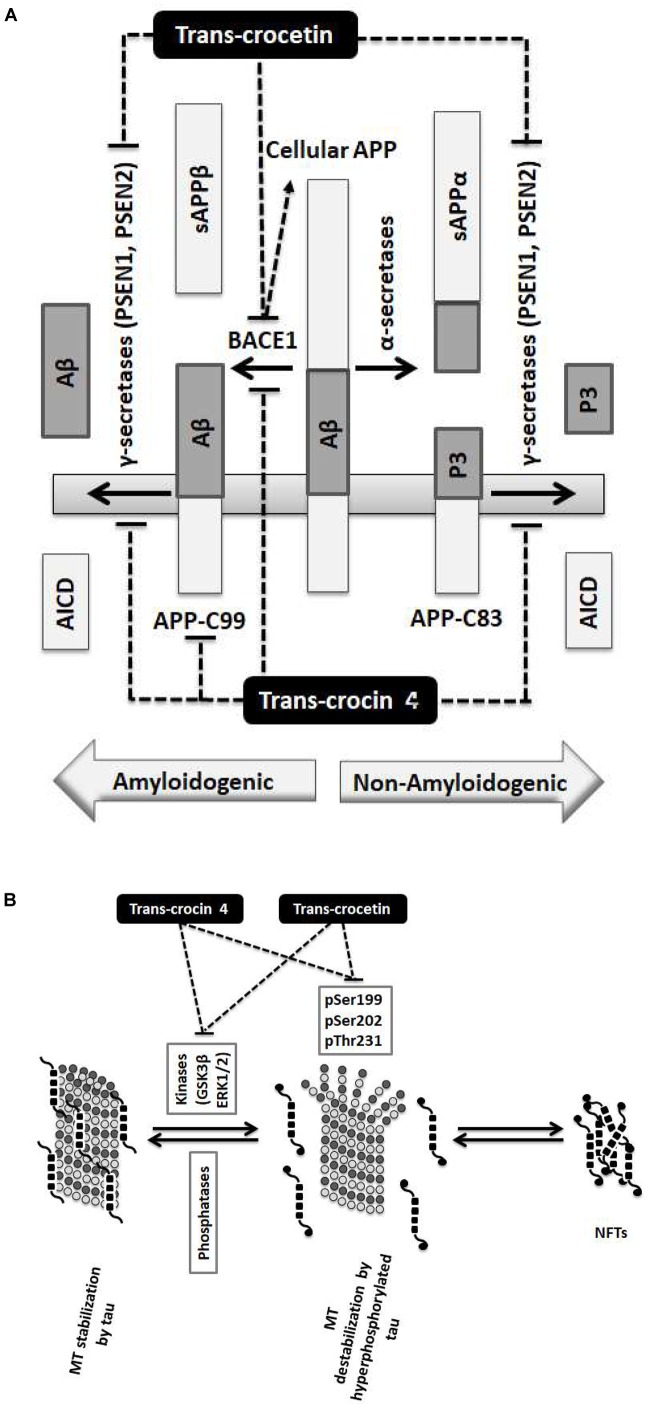
Summary diagram of the *trans*-crocin 4 and *trans*-crocetin effects on APP processing, and tau phosphorylation. **(A)** APP is processed by the non-amyloidogenic (alpha) or the amyloidogenic (beta) proteolytic pathways. Through the non-amyloidogenic pathway, APP is cleaved by the alpha secretases to produce soluble APP alpha (sAPPα) and APP-C83 in the plasma membrane. APP-C83 is cleaved by the gamma secretases (PSEN1 and PSEN2) to produce APP intracellular C-terminal domain (AICD) and P3 peptides. Through the amyloidogenic pathway, APP is cleaved by BACE1 (beta secretase) releasing sAPPβ to the extracellular space, and leaving APP-C99 in the plasma membrane. APP-C99 is subsequently processed by the gamma secretases into amyloid beta (Aβ) and AICD. Some species of Aβ, in particular Aβ_1-42_, Aβ_1-40_, and Aβ_3-40_, are particularly toxic forming protofibrils, annular assemblies, soluble toxic oligomers, and insoluble inert β-sheet amyloid fibers extracellularly. The accumulation of these fibers ultimately lead to synaptic failure, neuronal death and AD. *Trans*-crocin 4 and *trans*-crocetin block the amyloidogenic pathway in the SH-SY5Y-APP AD model by reducing the levels of BACE1 and gamma secretases. In addition, *trans*-crocin 4 reduces the levels of APP-C99. **(B)** Tau (shown as multiple black square units) is a microtubule (MT, shown as dark and light gray circles) associated protein enhancing their stabilization (left panel). Tau protein is phosphorylated at multiple sites by GSK3β and ERK1/2 kinases. Hyperphosphorylation of tau (shown as multiple black square units with black circles at their ends when phosphorylated) leads to its dissociation from microtubules, microtubule destabilization, loss of dendritic microtubules and synapses, interruption of axonal transport, plasma membrane degeneration, neuronal loss and AD development (middle panel). The hyperphosphorylated and misfolded tau forms intracellular fibrils and neurofibrillary tangles (NFTs) (right panel). *Trans*-crocin 4 and *trans*-crocetin block the formation of NFTs in the PC12-htau AD model by reducing the tau phosphorylation at multiple sites as well as the activities and levels of tau kinases.

Our findings demonstrate that the neuroprotective effects of *trans*-crocin 4 and *trans*-crocetin can be mediated by their significant modulation of multiple key steps in the APP processing and tau phosphorylation pathways (Figure 6). Notably, both *trans*-crocin 4 and *trans*-crocetin significantly affect both pathways in the AD cell culture models tested, SH-SY5Y-APP and PC12-htau, respectively, and their effects are largely overlapping. In specific, they both have a significant impact on the β- (BACE1) and γ-secretases (PSEN1 and PSEN2) by reducing their levels, and consequently serving toward the inhibition of the amyloidogenic pathway, as well as by reducing the activity and levels of tau kinases, reducing tau phosphorylation and ultimately favoring the inhibition the of NFTs formations. In conclusion, the molecular evidence we present for these two compounds together with previous phenotypic observations and the recent reports on *trans*-crocetin crossing the blood–brain-barrier (Lautenschlager et al., 2015), support their potential value in combating AD. Future studies will explore the functional effects of *trans*-crocin 4 and *trans*-crocetin *in vivo*.

## Author Contributions

IC, DA, NK, and AG performed the experiments. ZP-D and AT conceived the study. AT and AS provided the plant extracts and had critical input toward their utilization. DS co-ordinated and supervised the study. IC, DA, NK, AS, AT, and DS contributed to the critical evaluation/interpretation of the results and to the preparation of the manuscript.

## Conflict of Interest Statement

The authors declare that the research was conducted in the absence of any commercial or financial relationships that could be construed as a potential conflict of interest.

## Footnotes

1 https://imagej.nih.gov/ij/
