# Supplementary material for: The Crocus sativus Compounds trans-Crocin 4 and trans-Crocetin Modulate the Amyloidogenic Pathway and Tau Misprocessing in Alzheimer Disease Neuronal Cell Culture Models
Source: Front Neurosci. 2019 Mar 26;13:249. doi: 10.3389/fnins.2019.00249 (PMC6443833; doi:10.3389/fnins.2019.00249)
Supplement: Supplementary file 1 [file Data_Sheet_1.pdf]

The *Crocus sativus* compounds *trans*-crocin 4 and *trans*-crocetin modulate the amyloidogenic pathway and tau misprocessing in Alzheimer disease neuronal cell culture models

Ioanna Chalatsa[1†], Demetrios A. Arvanitis[2†], Nikolaos Stavros Koulakiotis[3], Athina Giagini[1], Alexios Leandros Skaltsounis[4], Zeta Papadopoulou-Daifoti[5‡], Anthony Tsarbopoulos[3,5], *Despina Sanoudou[1,2,6*]

Correspondence:

Despina Sanoudou, PhD FACMG

E-mail dsanoudou@med.uoa.gr

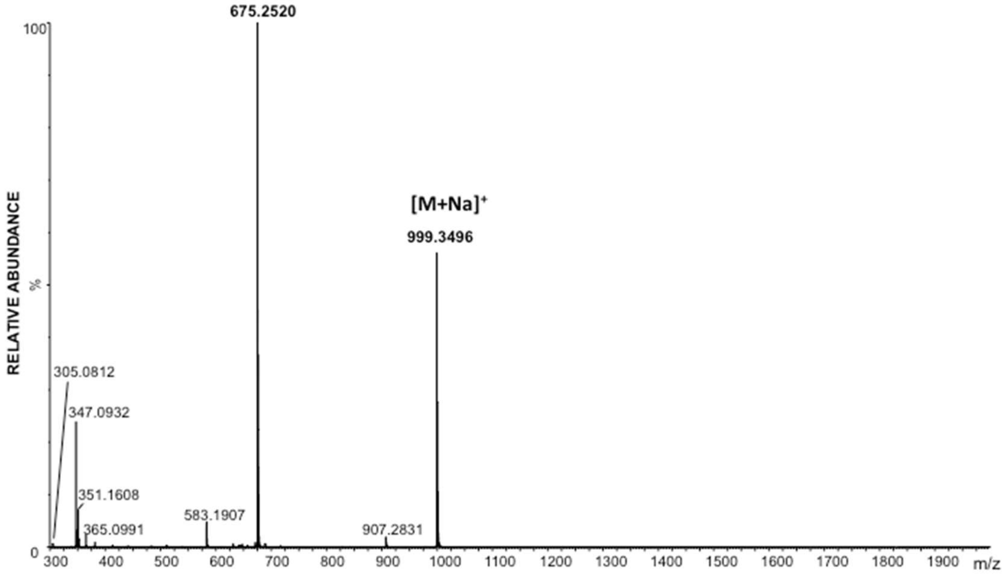

Supplementary Figure 1: Positive-ion ESI product ion spectrum of the $[M+Na]^+$ precursor ions of *trans*-crocetin utilizing a Waters Premier quadrupole reflectron time-of-flight (QqRTOF) mass spectrometer.

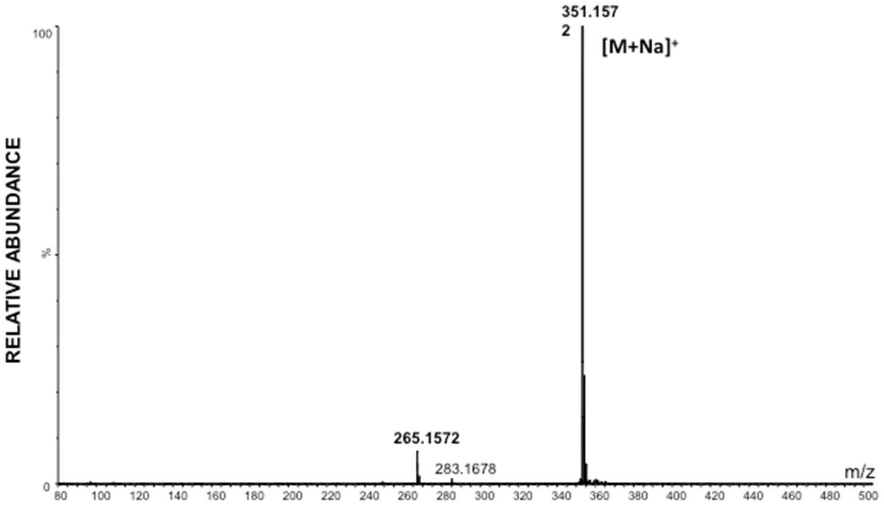

Supplementary Figure 2: Positive-ion ESI product ion spectrum of the $[M+Na]^+$ precursor ions of *trans*-crocin-4 utilizing a Waters Premier quadrupole reflectron time-of-flight (QqRTOF) mass spectrometer**.**

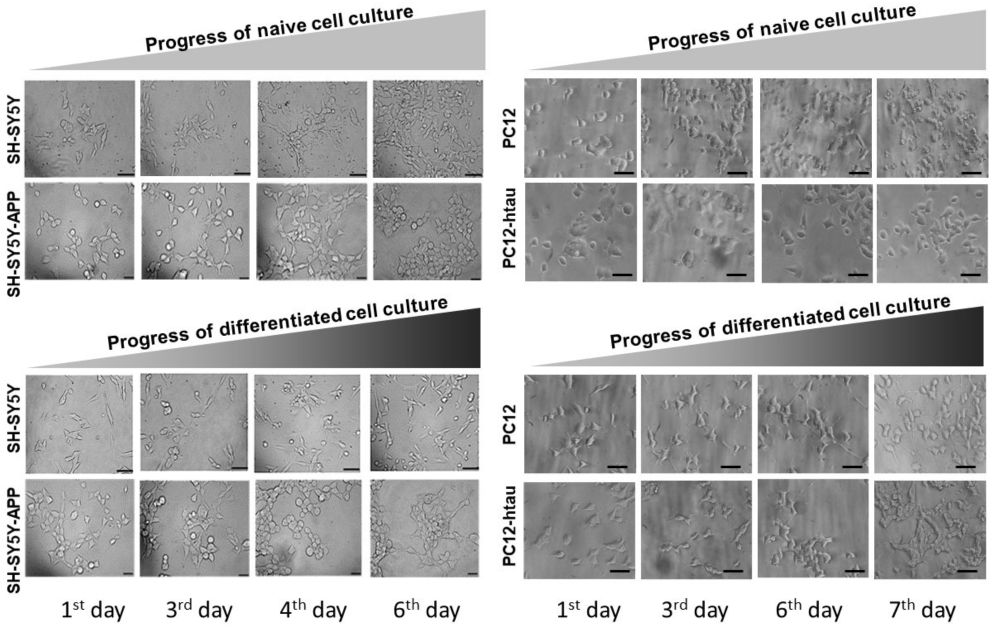

Supplementary Figure 3: Differentiation of SH-SY5Y, SH-SY5Y-APP, PC12 and PC12-htau to neuron-like cells. Representative photos of SH-SY5Y and SH-SY5Y-APP (A), PC12 and PC12-htau naive cells (B) at 6 and 7 days, respectively, of proliferation and differentiation (C and D). Differentiated cells exhibit decreased proliferation, and increased dendritic and neuritic-like projections compared to naive cells.

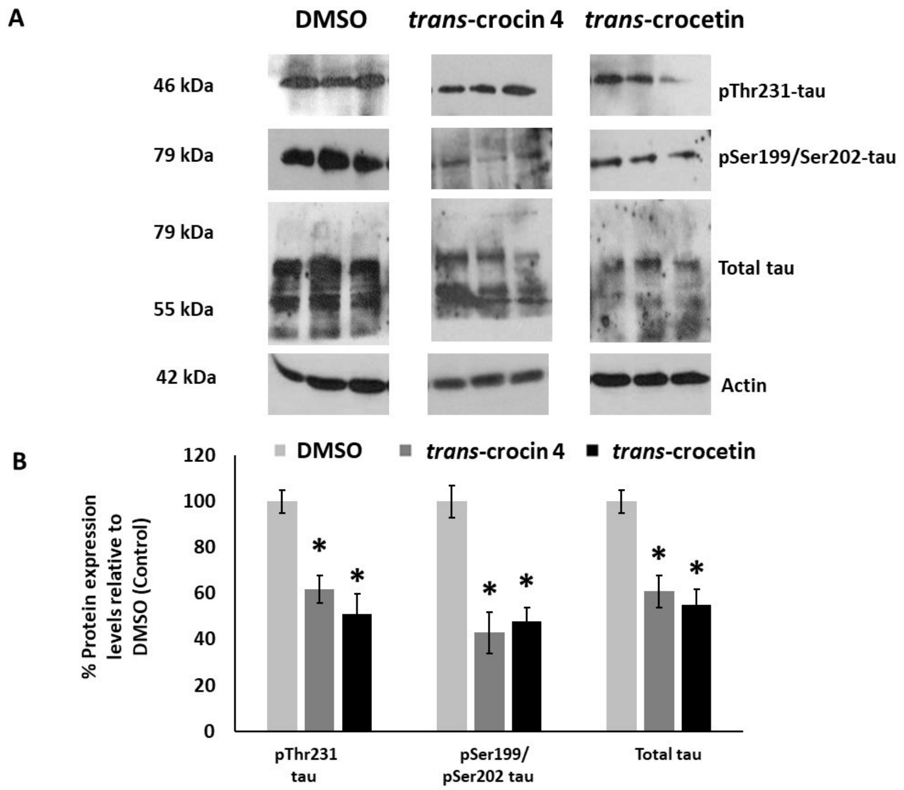

Supplementary Figure 4: Immunoblotting assessment of the tau levels and phosphorylation after treatment of differentiated PC12 cells with *trans*-crocin 4 or *trans*-crocetin. (**A**) Immunoblotting detection of pThr231-tau, pSer199/Ser202-tau, total tau, with actin as internal control of protein expression. (**B**) Diagrammatic presentation of quantified protein expression (* $p<0.05$, when compared test treatment versus DMSO in post-hoc one-way ANOVA, n=3).

Supplementary Table 1: Primary antibodies and electrophoresis conditions

Protein	Catalogue number	Company	MW	Host	Gel
**Cellular APP**	R1(57)	By Prof. Efthimiopoulos Lab	~110 kDa	Polyclonal anti-rabbit	10% Tris-Glycine
**APP-C99**	R1(57)	By Prof. Efthimiopoulos Lab	12 kDa	Polyclonal anti-rabbit	4-12% Bis-Tris
**APP-C83**	R1(57)	By Prof. Efthimiopoulos Lab	10 kDa	Polyclonal anti-rabbit	4-12% Bis-Tris
**sAPPα**	SIG-39320	Covance	~100 kDa	Monoclonal anti-mouse	16.5% Tris-Tricine
**beta-amyloid**	SIG-39320	Covance	4 kDa	Monoclonal anti-mouse	16.5% Tris-Tricine
**BACE1**	MAB5308	Chemicon Millipore	56 kDa	Monoclonal anti-mouse	10% Tris-Glycine
**PSEN1**	AB5308	Chemicon Millipore	Multiple forms	Polyclonal anti-rabbit	10% Tris-Glycine
**PSEN2**	2192	Cell Signaling	Multiple forms	Polyclonal anti-rabbit	10% Tris-Glycine
**Total tau (Tau5 antibody)**	ab80579	Abcam	79 kDa	Monoclonal anti-mouse	10% Tris-Glycine
**pSer199/Ser202-tau**	AB9674	Chemicon Millipore	79 kDa	Polyclonal anti-rabbit	10% Tris-Glycine
**pThr231-tau**	ab151559	Abcam	46 kDa	Monoclonal anti-rabbit	10% Tris-Glycine
**pERK1/2**	4370	Cell Signaling	44/42 kDa	Monoclonal anti-rabbit	10% Tris-Glycine
**ERK1/2**	4695	Cell Signaling	44/42 kDa	Monoclonal anti-rabbit	10% Tris-Glycine
**pSer9-GSK3β**	9323P	Cell Signaling	46 kDa	Monoclonal anti-rabbit	10% Tris-Glycine
**GSK3β**	9315S	Cell Signaling	46 kDa	Monoclonal anti-rabbit	10% Tris-Glycine

**Actin**	MAB1501	Chemicon<br>Millipore	42 kDa	Monoclonal anti-mouse	
**GAPDH**	SC-365062	Santa Cruz	37 kDa	Monoclonal anti-mouse	
